# Signaling pathways involved in the biological functions of dendritic cells and their implications for disease treatment

**DOI:** 10.1186/s43556-023-00125-3

**Published:** 2023-05-15

**Authors:** Hao Cheng, Wenjing Chen, Yubin Lin, Jianan Zhang, Xiaoshuang Song, Dunfang Zhang

**Affiliations:** Department of Biotherapy, State Key Laboratory of Biotherapy and Cancer Center, Collaborative Innovation Center of Biotherapy, West China Hospital, Sichuan University, Chengdu, 610041 Sichuan China

**Keywords:** Dendritic cells, T cell, Immunotherapy, Homeostasis, Tumor, Autoimmune diseases

## Abstract

The ability of dendritic cells (DCs) to initiate and regulate adaptive immune responses is fundamental for maintaining immune homeostasis upon exposure to self or foreign antigens. The immune regulatory function of DCs is strictly controlled by their distribution as well as by cytokines, chemokines, and transcriptional programming. These factors work in conjunction to determine whether DCs exert an immunosuppressive or immune-activating function. Therefore, understanding the molecular signals involved in DC-dependent immunoregulation is crucial in providing insight into the generation of organismal immunity and revealing potential clinical applications of DCs. Considering the many breakthroughs in DC research in recent years, in this review we focused on three basic lines of research directly related to the biological functions of DCs and summarized new immunotherapeutic strategies involving DCs. First, we reviewed recent findings on DC subsets and identified lineage-restricted transcription factors that guide the development of different DC subsets. Second, we discussed the recognition and processing of antigens by DCs through pattern recognition receptors, endogenous/exogenous pathways, and the presentation of antigens through peptide/major histocompatibility complexes. Third, we reviewed how interactions between DCs and T cells coordinate immune homeostasis *in vivo via* multiple pathways. Finally, we summarized the application of DC-based immunotherapy for autoimmune diseases and tumors and highlighted potential research prospects for immunotherapy that targets DCs. This review provides a useful resource to better understand the immunomodulatory signals involved in different subsets of DCs and the manipulation of these immune signals can facilitate DC-based immunotherapy.

## Introduction

Dendritic cells (DCs), as key outposts of the immune response, are crucial for maintaining the central and peripheral tolerance mechanisms under steady-state conditions. Alternatively, under inflammatory conditions, danger signals from sites of infection or cancer promote the recruitment, activation, and maturation of DCs, ultimately leading to antigen-specific T cell responses [1, 2]. The migration of DCs towards lymph nodes and their upregulation of co-stimulatory molecules, such as CD40, CD80, and CD86, as well as secretion of multiple cytokines, including interferon (IFN)-α/β, interleukin (IL)-10, and IL-12, are induced by danger signals [3, 4]. These events determine the activation and polarization of T cells. Owing to their role in initiating T cell immunity, DCs have been identified as key regulators in guiding immunotherapy for autoimmune diseases and tumors [3, 5]. Existing evidence indicates that the presence of DCs or expression of DC-specific transcriptional signatures positively correlates with CD8^+^ T cell infiltration in tumors and improved prognosis, while selective depletion of DCs reduces the number of T cells in autoimmune diseases and attenuates disease severity [6–9].

The biological functions and markers of DCs may vary considerably in different microenvironments; this increases the difficulty of classifying DCs. Currently, a uniform classification system is to divide DCs into conventional DCs (cDCs), plasmacytoid DCs (pDCs), and monocyte-derived DCs (moDCs) based on their origin and differentiation pathways. Over the past two decades, research surrounding DCs has yielded extraordinary insights into various aspects of biological and medical applications. Nonetheless, these advances have highlighted many fundamental unanswered questions surrounding the function and mechanisms of DCs. Addressing these questions can accelerate the translation of this acquired knowledge on DCs into corresponding clinical applications. Therefore, the key aims of this review are to provide an overview of the molecular signals that control DC differentiation, antigen presentation, and immune regulation, and to highlight the potential applications of targeting DCs for immunotherapy. We also discuss the advancements in understanding how DCs crosstalk with other immune cells, as well as their role in shaping the tumor microenvironment. Overall, this review aims to comprehensively evaluate the signaling pathways that are involved in DC biology and their potential implications in disease treatment.

## Developmental and biological functions of DC subsets

In 1973, Steinman and Cohn described a type of stellate cell structure in mouse spleens and lymph nodes, and termed them DCs [10]; prior to which macrophages and B cells were considered the primary antigen-presenting cells (APCs) [11]. Their experiments over the next few years confirmed that DCs are far more potent as APCs in stimulating T cell responses than other cell types that express the major histocompatibility complex (MHC) and co-stimulatory molecules [12]. DCs are present in almost all tissues throughout the body and exhibit a high degree of heterogeneity. Different DC subpopulations are defined based on their development, anatomical location, and immunological characteristics.

### Development and transcriptional regulation of DC subsets

**Fig. 1:**
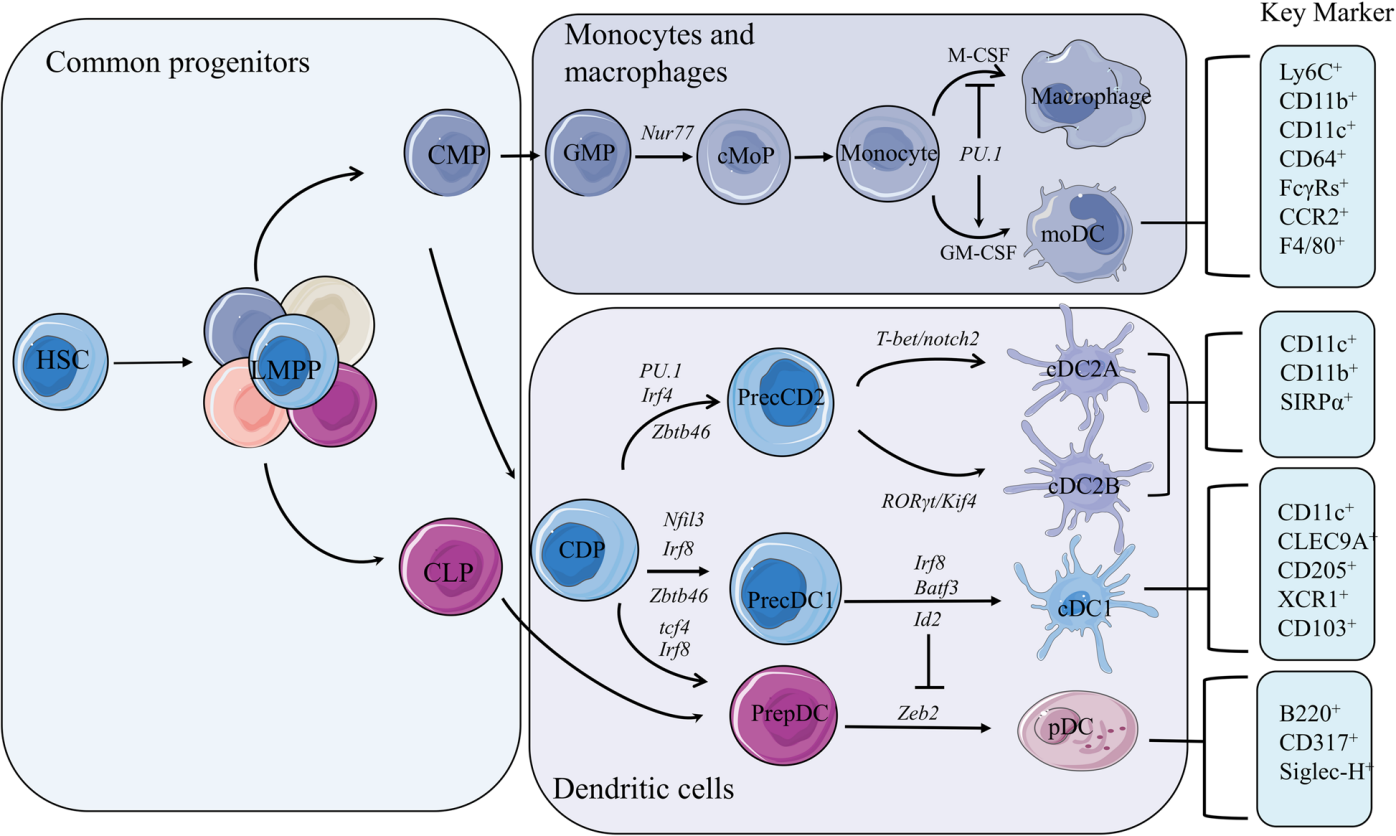
Mouse dendritic cell development and lineage. DCs are derived from CDP, which is derived from LMPPs differentiated from HSC in the bone marrow. Branching CMPs of LMPPs are driven by the transcription factor Nuf77 to differentiate into monocytes and then further differentiate into M-CSF-dependent macrophages or GM-CSF-dependent moDCs. High expression of PU.1 is associated with the specification of these two cell types. Zbtb46 expression in pre-cDC1s and pre-cDC2s is a specific marker for the specification of cDCs. cDC2 subsets express high levels of IRF4, whereas pDCs and cDC1 express high levels of IRF8. Id2 is a suppressor of Tcf4, impairing Tcf4-driven pDCs specification and promoting cDC1s specification. HSC, hematopoietic stem cell; LMPP, lymphoid- primed multipotent progenitors; CLP, common lymphoid progenitors; GMP, granulocyte–macrophage progenitors; cMoP, common monocyte progenitors; IRF8, interferon regulatory factor 8; BATF, basic leucine zipper ATF-like transcription factor; SIRPα, signal regulatory protein alpha

DCs originate from common myeloid progenitors (CMPs). Their main types include cDCs, pDCs, and moDCs (Fig. 1). In certain inflammatory conditions, the transcription factor *Nur77* is responsible for promoting the differentiation of CMP into monocytes, which subsequently develop into moDCs [13, 14]. cDCs and pDCs are considered to have originated from a shared lineage of DC precursors known as common DC precursors (CDPs), which lack monocyte/macrophage differentiation potential and also develop from CMPs [15]. CDPs are typically characterized by a lack of lineage markers (LIN^−^) and are accompanied by the expression of Fms-like tyrosine kinase 3 (FLT3), macrophage colony-stimulating factor receptor (M-CSFR; also known as CD115), and receptor tyrosine kinase KIT (SCFR; also known as CD117) [16]. The transcription factor Zbtb46 is a specific marker for cDCs, which comprises two main subsets: the cDC1s and the more heterogeneous cDC2s [17].

#### cDC1 and cDC2 subsets

cDC1s and cDC2s play special roles in the immune activation of CD8^+^ and CD4^+^ T cells, respectively, through differential processing and presentation of antigens and the production of corresponding cytokines [18, 19]. In the bone marrow, upregulation of CD11c expression facilitates CDPs to further develop into pre-cDCs, which are excreted from the bone marrow via the blood and differentiated into cDC1 and cDC2 populations in tissues [11, 20]. cDC1s develop from pre-DCs in an IRF8-dependent manner, with cell surface expression of the chemokine receptor XCR1, C-type lectin–like receptor (CLEC9A), and CD205 [21–23]. In addition, Nfil3 and Id2 are essential for the differentiation of cDC1s. Selective deletion of cDC1s occurs in mice with *Batf3*, *Nfil3*, or *Id2* knockout [4]. Interestingly, the effects of deletions in *Batf3*, *Id2*, or *Nfil3* in mice can be repaired by ectopic expression of IRF8, suggesting that high expression of IRF8 is central to cDC1 development [24]. Similarly, the compensatory effects of other members of the BATF family maintain IRF8 expression under some inflammatory conditions, prompting to cDC1 production in Batf3^−/−^ mice [25].

Compared to cDC1s, cDC2s have a different transcriptional profile and can be identified by the expression of SIRPα and CD11b, with the key transcription factor being IRF4 [11]. Although cDC2s are present in IRF4^−/−^ mice, their numbers are substantially reduced and they lose the ability to migrate peripherally to the lymph nodes [26, 27]. In addition, the KLF4 transcription factor and Notch2 receptor are essential for the development of cDC2s [28, 29]. cDC2s are further divided into several subsets based on their phenotype and function owing to their high heterogeneity. High expression of the endothelial cell-selective adhesion molecule (ESAM) has been used to distinguish Notch2-dependent cDC2s (ESAM^hi^ cDC2s) [30]. ESAM^hi^ cDC2s have a high value-added rate and high expression of MHCII compared to the ESAM^lo^ cDCs, which preferentially express monocyte-associated genes, including chemokine (Ccr2) receptors and lysozyme (Lyz1, Lyz2) [30]. Brown et al. defined the two subpopulations of cDCs: cDC2sA and cDC2sB, based on the mutually exclusive expression of the transcription factors *T-bet* or *RORγT *[31]. The cDC2sA population corresponds, at least in part, to the previously identified ESAM^hi^ cDC2s. cDC2sB has a phenotype similar to that of ESAM^lo^ cDC2s. T-bet^+^ cDC2sA are present throughout lymphoid and non-lymphoid tissues, in contrast to ESAM^hi^ cells, which are found only in the spleen and mesenteric lymph nodes [32].

Human and mouse cDCs are thought to be relatively evolutionarily conserved. They are both derived from CDPs and comprise mainly two basic subtypes, cDC1s and cDC2s. Similar to mouse cDCs, the development of human cDC1s’ depends on Batf3 and IRF8 expression. Short hairpin RNA-mediated knockdown of Batf3 selectively inhibits the development of human cDC1s in vitro [33]. Patients with IRF8 mutations were found to have defective helper T cell function, likely due to reduced CD11c-expressing DCs, and had reduced resistance to infections, including Mycobacterium infections [34]. Human cDC1s were further identified by the expression of CD141 and CADM1 in addition to the expression of the mouse cDC1s-specific markers XCR1 and CLEC9A. In contrast, human cDC2s are commonly identified by the expression of CD1c (BDCA1) and CLEC10A (CD301a), in addition to SIRPα [35]. In a recent study, single-cell RNA-sequencing analysis was used to classify human cDC2s into DC2 and DC3 and was validated by parallel observations in their murine cDC2s homologs, including ESAM^hi^ cDC2s and ESAM^lo^ cDC2s [36].

#### pDC subsets

pDCs are derived from CDPs and are consistently produced in the bone marrow. They, then, mature and migrate to the periphery, where they lose their proliferative ability and only survive for a short time [37]. However, a portion of pDCs may originate from the lymphoid lineage, as evidenced by the discovery of a population of LIN^−^KIT^+^SCA1^+^CD34^+^FLT3^+^ cells that appear to be directly derived from lymphoid-primed multipotent progenitors; this population expresses high levels of Tcf4 with prominent pDC differentiation potential [38]. The pDCs possess elevated levels of Toll-like receptors (TLRs) that are capable of sensing endosomal nucleic acids, such as TLR7 and TLR9 [39, 40]. These receptors enable pDCs to recognize viral single-stranded RNA and unmethylated CpG motif-containing DNA [41]. FLT3 and its ligand (FLT3L) play crucial roles in the development of pDCs [42]. FLT3L drives spontaneous differentiation of pDCs and lineage bifurcation of pDCs and cDCs through the activation of signal transducer and transcription 3 (STAT3) and mammalian target of rapamycin (mTOR) [43]. Subsequent specification of pDCs requires the involvement of E protein transcription factor TCF4 (E2-2), which is a key factor in the identification of pCDs in mice and humans. [44]. Id2, a repressor of E2-2, may disrupt the E2-2-driven transcriptional program of pDCs, directing cell differentiation towards cDC1s [16]. Thus, Id2 is essentially absent in pDCs and relatively highly expressed in cDC1s. The numbers of pDCs are increased in Id2^-/-^ mice. Overexpression of Id2 or de-repression of Id2 caused by the loss of the transcriptional repressor ZEB2 results in impaired development of pDCs and enhanced development of cDC1s [45–47]. In conclusion, these studies reveal a complex regulatory network for the development of pDCs and highlight the close relationship between the genetic mechanisms involved in the specification of cDCs and pDCs.

#### moDC subsets

In mice, two subsets of CMP-derived mononuclear phagocytes exist: CCR2^−^CX3Cr1^hi^ LY6C^−^ and CCR2^+^CX3Cr1^lo^LY6C^+^CD62L^+^; the latter is a precursor to moDCs [48]. In contrast to the steady state, cDC2s express moDC-specific markers such as Ly6C, CD64, and MAR1 in the inflammatory microenvironment, which makes the identification of moDCs challenging [49]. To address this, Guilliams et al. compiled the available gene expression data for DCs and macrophage subsets. The findings showed that the expression of Fc receptors for IgG (FcγRs) is highly selective in humans and mice [50]. Specifically, moDCs highly express most activating and inhibitory FcγRs, while cDCs and pDCs predominantly express inhibitory FcγRIIB [50]. Of note, LY6C^+^ monocytes can differentiate into iNOS^+^ microbicidal macrophages in addition to CCR2-dependent moDCs [51, 52]. Menezes et al. suggested that PU.1 is critical in regulating the polarization of Ly6C^+^ monocytes and that high levels of PU.1 can selectively promote GM-CSF-dependent moDC production and downregulate the production of iNOS^+^ macrophages [53]. Furthermore, the aryl hydrocarbon receptor AHR serves as a molecular switch that plays a role in determining the fate of monocytes. In the presence of macrophage colony-stimulating factor (M-CSF), monocytes preferably differentiate into macrophages, whereas in the presence of IL-4 and TNF-α together with AHR ligands, they tend to differentiate into moDCs [54]. Overall, inflammatory monocytes have multiple fates, and the precise conditions in the microenvironment that drive the differentiation of monocytes towards moDCs rather than macrophages, as well as the specific molecular coordinators involved, remain to be further defined.

### Antigen uptake and presentation by DC cells

In the previous section, the development and origin of the cDC, pDC, and moDC subsets were discussed. However, why these cells constitute the DC population is unclear. All of these cells bridge natural and adaptive immunity, including sensing the tissue environment, processing and presenting antigens, and promoting T cell responses, even though the pDC and moDC subsets are far less capable of initiating T cell responses compared to cDCs. Resting state DCs, are considered immature and must undergo an intricate and rigorous series of antigen-acquisition processes prior to activation and maturation[55]. Mature DCs exhibit reduced phagocytosis, increased antigen presentation and migration, and upregulated expression of various co-stimulatory molecules including CD40, CD70, and CD86, as well as MHC class I and class II molecules [40, 56].

#### Molecular players in antigen recognition and internalization

Interaction of pattern recognition receptors (PRRs) with pathogen-associated molecular patterns (PAMPs) or damage-associated molecular patterns is a prerequisite for initiating immune responses in DCs. In mice and humans, the PRR families include TLRs, retinoic acid-inducible gene-I-like receptors (RLRs), nucleotide oligomerization domain (NOD)-like receptors (NLRs), C-type lectin receptors (CLRs), and range of intracellular DNA sensors [57–59]. High expression of TLR3, which senses viral dsRNA, is conserved in human and mouse cDC1s. TLR3 specifically initiates the efficient cDC1-mediated clearance of infected cells by CD8^+^ T cells [11, 60, 61]. Although the generation of a robust type I IFN response in cDC1s is biased towards TLR3, TLR9 activation is equally able to regulate Myd88-IRF7 signaling in cDC1s via NCoR1, inducing a moderate antiviral response [62, 63]. Additionally, mouse cDC1s express TLR11 and TLR13, whereas human cDC1s highly express TLR8[14, 64, 65]. Unlike cDC1s, cDC2s rapidly accumulate in mesenteric lymph nodes in a TLR5-dependent manner after soluble flagellin immunization [66]. cDC2s also express TLR2, TLR4, and TLR7 for sensing bacterial and viral associated PAMPs [36, 67]. The pDC-mediated antiviral response is largely dependent on the activation of TLR9 signaling and subsequent Myd88-IRF7 signaling[68]. In addition, TLR7 is responsible for the induction of inflammatory signaling following SARS-CoV-2 infection in a stem cell–based human pDC model [69]. Meanwhile, endosomal TLRs are responsible for initiating the initial steps in the generation of type I IFN in pDCs, whereas RLRs contribute to the latter stages of the IFN response [58, 70].

RLRs are constitutively expressed in cDCs and are the primary molecules used by cDCs to sense foreign nucleic acids [71]. As cytoplasmic sensors of viral RNA, RLRs interact with an adaptor protein (MAVS) upon activation, thereby triggering a signaling cascade that includes the secretion of inflammatory cytokines and chemokines [72, 73]. The calcium-dependent carbohydrate recognition domain in CLRs recognizes glycans in the fungal cell wall and is central to the body’s resistance to fungal infections [74]. CLR activation recruits Syk to initiate downstream signaling, leading to the production of ROS and activation of the NF-κB pathway mediated by the CARD9/Bcl-10/MALT1 complex [75, 76]. Expression of one CLR members, DNGR-1 (encoded by CLEC9A), is largely restricted to cDC1s, which promote phagosome rupture and cross-presentation of dead cell-associated antigens via the DNGR-1 pathway [77, 78]. When annexin A2, a protein commonly found in individuals with nasopharyngeal carcinoma, interacts with CLRs, an immunosuppressive response and release of IL-10 from DCs can be triggered [79].

**Fig. 2:**
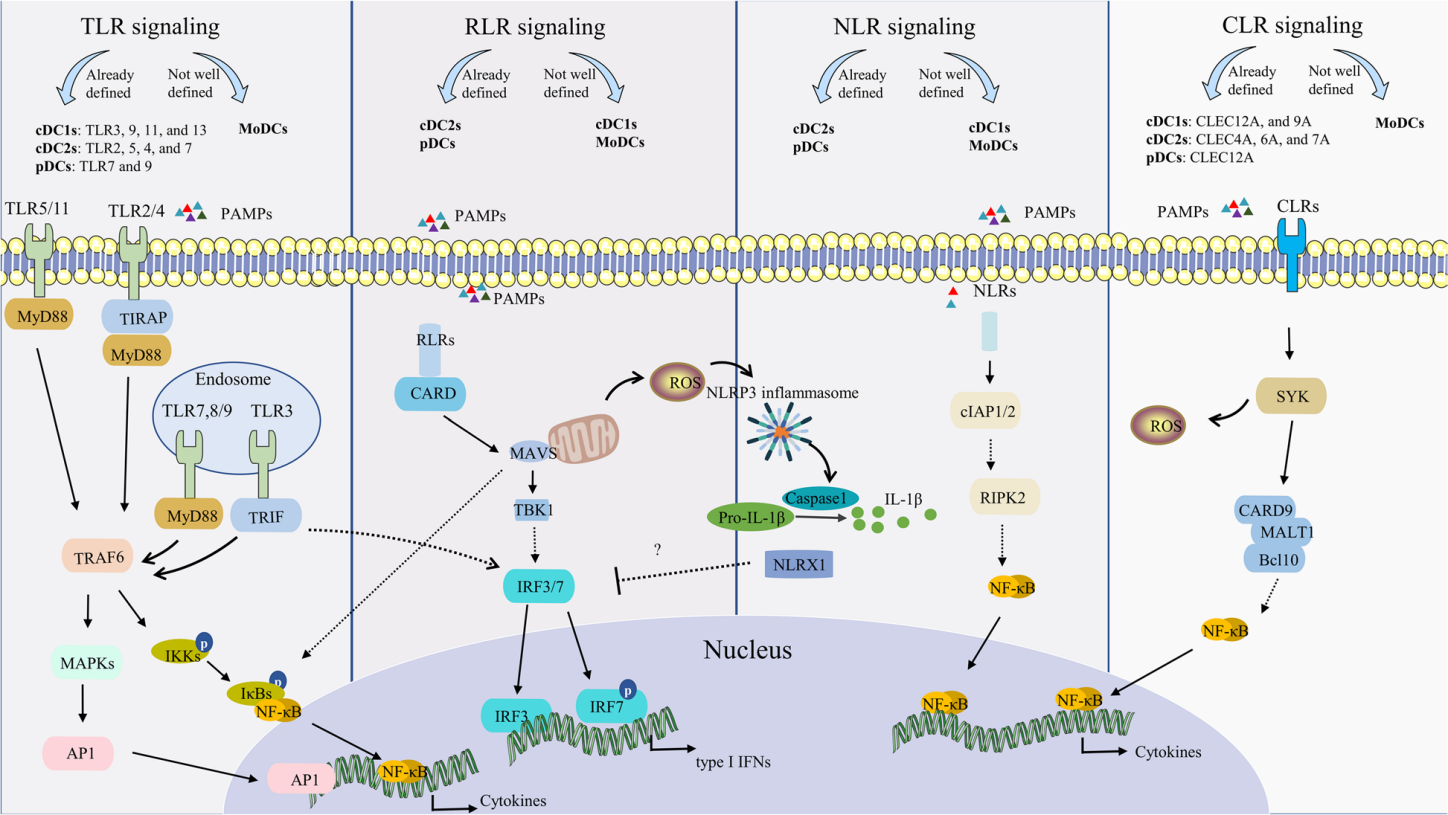
PRRs of DCs and their signaling pathways. The four major subgroups of DCs (cDC1s, cDC2s, pDCs, and moDCs), each have distinct PRRs. TLR2, TLR4, TLR5, TLR7, TLR8, TLR9, and TLR11 activate TRAF6 predominantly through Myd88; this then leads to the activation of IKKs and the release of NF-κB for nuclear translocation. In addition, TRAF6 can promote AP1 activity through MAPK signaling [58]. TLR3 signaling is primarily mediated by TRIF. TLR3 can promote the activation of TRAF6 and IRF3/IRF7. Activated RLRs interact with MVAS through their signaling structural domain, CARD, to activate TBK1, which then phosphorylates IRF3 and IRF7, translocating them from the cytoplasm to the nucleus [73]. In addition, the RLR signaling pathway activates NLRP3 inflammatory vesicles, which promote the formation of IL-1β. When NLR receptors are activated, they aggregate and activate downstream signaling molecules, including RIPK2, which in turn triggers the activation of NF-κB [58]. CLR activation recruits multiple kinases, including sky and the CARD9/Bcl10/MALT1 (CBM) complex, ultimately leading to activation of NF-κB [59]. TRAF6, TNF receptor associated factor 6; TRIF, TIR domain-containing adapter-inducing interferon-beta; ROS, reactive oxygen species; CARD, caspase recruitment domain; TBK1, TANK-binding kinase 1; RIPK2, receptor-interacting serine/threonine protein kinase 2

Increasing evidence indicates that different members of the PRRs family can counteract pathogens by interacting with each other or attenuating excessive inflammation by mutual antagonism [80, 81]. Kim et al. showed that the collaboration between TLR2 and NOD2 is required for the induction of pro-IL-1β and NOD-like receptor pyrin domain-containing 3 (NLRP3) in *Helicobacter pylori*-infected murine-derived DCs [82]. In addition, NLRX1, a member of the NLR family, negatively regulates RLR-mediated type I IFN production in human pDCs [71]. In conclusion, the differences in intrinsic immune receptor expression between different subsets of DCs confer the corresponding ability of DCs to respond appropriately in response to different pathogens and stress signals (Fig. 2).

As tissue sentinels, DCs take up antigens through phagocytosis, micro- or macropinocytosis, and endocytosis [40]. After acquisition by DCs, the antigen is processed either by an endogenous or exogenous pathway. In general, antigens processed by the endogenous pathway are bound to MHC I molecules and presented to CD8^+^ cells, whereas antigens processed by the exogenous pathway are bound to MHC II molecules and presented to CD4^+^ cells [4, 40]. However, DCs are also capable of presenting peptides of exogenously internalized antigens via MHC I molecules, a process known as ‘cross-presentation’ [83]. Autophagy protein-mediated degradation of extracellular material provides substrates for MHC class II presentation to late endosomes and lysosomes [84, 85]. Conditional knockdown of the autophagy protein ATG5 in cDCs reportedly resulted in mice that were completely resistant to the development of experimental autoimmune encephalomyelitis (EAE) and had significantly reduced CD4^+^ T cell aggregation in the central nervous system [86]. DCs possess fewer proteases and are more susceptible to modulation by pH, unlike macrophages. Thus, they degrade internal antigens slowly but more efficiently recover immunogenic peptides and assemble peptide-MHC II complexes [87]. Reactive oxygen species (ROS), generated by the NADPH oxidase (NOX) complex or by electron leakage from mitochondrial aerobic respiration, promote alkalinization of phagosomal pH and inhibit rapid antigen degradation by acidic lysosomal proteases [88]. In contrast, activation of the transcription factor TFEB in mouse DCs induces a reduction in lysosomal pH and elevation in lysosomal protease expression, which results in downregulation of processing and presentation by MHC class II antigens and upregulation of processing and presentation by MHC class II antigens [89, 90]. In addition, the immunosuppressive function of the anti-inflammatory cytokine IL-10 is because of its ability to reduce the expression of MHC II molecules [91].

#### Antigen presentation

DCs are the predominant cell population responsible for presenting antigens in vivo and induce the activation of antigen-specific T cells through the interaction of the antigenic peptide-MHC complex with TCRs in combination with appropriate co-stimulatory signals. cDC1s highly express MHC class I molecules and possess a superior cross-presentation capability, which are necessary for CD8^+^ T cell activation. The binding of DNGR-1 to F-actin in cDCs exposed to dead cell debris facilitates cross-presentation of relevant antigens in tumors. Therefore, abundant DNGR-1 is positively correlated with the survival of cancer patients [92–94]. Secreted gelsolin blocks DNGR-1-dependent cross-presentation and inhibits antitumor CD8^+^ T-cell responses [92]. In contrast, WD Repeat- and FYVE domain-containing protein 4 (WDFY4)-regulated vesicular transport pathway is essential for DNGR-1 to bind dead cell antigens and facilitate cross-presentation [95]. TLR stimulation also upregulates the cross-presentation of cDC1s, which is associated with a reduction in Rab34-dependent phagolysosome fusion[96]. Earlier, pDCs were not considered cross-presenting DCs. However, a plethora of evidence indicates that pDCs can activate CD8^+ ^cells by cross-presenting antigens in humans and mice [88, 97, 98]. Unlike cDC1s, the ability of pDCs to cross-present and activate CD8^+^ T cell responses are mainly dependent on mitochondrial ROS production rather than NOX1/2-mediated ROS production [88]. moDCs can also cross-present cell-associated antigens by using a different transcriptional program for cDCs [99].

DC-derived exosomes (Dex) are nanosized membrane vesicles that are initially formed by the inward budding of the endosomal membrane of DCs and subsequent release via the cell membran [90, 100]. Like DCs, Dex carries functional peptide-MHC complexes, co-stimulatory molecules (CD80 and CD86), and a variety of surface membrane proteins (integrins and ICAMs) that interact with immune cells [74, 100]. As a result, Dex can initiate antigen-specific CD4^+^ and CD8^+^ T cell responses directly or indirectly through antigen uptake and presentation. Exosomes derived from alpha-fetoprotein (AFP)-expressing DCs activate specific CD8 antitumor immune responses in tumor tissues [101]. Although Dex stimulate T cells in vitro and promotes increased secretion of IFN-γ and IL-2, these exosomes appear to be effective only for activated T cells, memory T cells, and T cell lines and are inefficient for the development of naïve T cells [101–103]. Compared to directly activating T cells with antigen-specific signals, the Dex antigen presentation pathway can stimulate T cell responses more efficiently by utilizing bystander DCs as intermediaries. One mechanism involves the direct transfer of peptide-MHC complexes to bystander DCs following the binding of Dex to the cell membrane of bystander DCs. Another possible mechanism is that Dex binds to bystander DCs and are reprocessed by the endosomal pathway, followed by the transfer of antigen epitopes of these Dex MHC molecules on bystander DCs for presentation to T-cells [102, 104].

## DCs maintain immune homeostasis through multiple signaling pathways

Although DCs are classified as innate immune cells because of their ability to recognize danger signals via multiple PRRs, as APC, they also play an important role in initiating the subsequent adaptive immune response. DCs provide a constant supply of antigens to T cells under infectious/inflammatory and steady-state conditions [105, 106]. Under noninflammatory conditions, DCs can present self-antigens derived from local tissues to T cells without inducing active autoimmunity [107]. These findings strongly suggest that DCs play a role in inducing peripheral immune tolerance and maintaining immune homeostasis.

### DC-mediated T cell homeostasis

The specification of an appropriate T cell fate is an important aspect of the effective adaptive immune function of T cells. For example, CD8^+^ cytotoxic T lymphocytes (CTL) kill infected or tumor cells, Th1 cells counteract intracellular bacterial and protozoal immune responses, Th2 cells respond to parasitic infections of the body, and Th17 cells counteract extracellular bacteria, and mycobacteria. DCs induce antigen-specific activation and expansion of T cells through peptide-MHC complexes and co-stimulatory signals, Expression of IRF4 by cDC2s is critical for the activation of CD4^+^ T cells; this may be attributed to the ability of IRF4 in enhancing the formation of peptide-MHC II complexes in cDC2s [4, 108]. DCs similarly instruct the fate of CD4^+^ cell polarization during antigen presentation (Fig. 2).

cDC1s are a major source of IL-12 in vivo and IL-12 signaling is essential for Th1 cell differentiation in mice and humans [109–111]. The absence of cDC1s in parasitic infection models such as *Trichuris muris* and *Leishmania major* infections is associated with impaired Th1 responses, suggesting that certain protective Th1 responses require the support of cDC1s in vivo [110, 112]. Unlike cDC1s, cDC2s adept at inducing Th2 and Th17 responses and are required for Th2 cell differentiation in the skin, lung, and intestine [113–115]. In particular, in the lungs of mice following house-dust extract inhalation, the partially mature Ly6C^+^ cDC2s lack CCR7 expression and promote Th17 differentiation, whereas the more mature CD200^+^ cDC2s expresse CCR7 and induce Th2 differentiation [116]. Tussiwand et al. suggested that differentiation of Th2 cells driven by cDC2s is dependent on the transcription factor KLF4 [28]. Conditional deletion of KLF4 in cDC2s impairs the Th2 cell response, but not Th1, Th17, or CTL responses [28]. The IL-13-induced signaling pathway promotes the STAT6-dependent differentiation of cDC2s and, subsequently, Th2 cells in the mouse dermis, while also reducing Th17 cell responses [117]. In humans, activation of formyl peptide receptor 3 (FPR3) enhances cDC2-mediated Th2 responses by decreasing IL-12 expression [118]. Interestingly, cDC1s have been recently found to similarly promote Th2 differentiation via p38α signaling [119]. TNFR2-cDC2s can promote the body’s Th1/17 immune response by activating moDCs after intranasal immunization with mucosal adjuvants [120]. Th17 cells play a key role in host defense and mucosal tissue homeostasis and are drivers of a variety of autoimmune diseases [121–123]. Furthermore, cDC2s provide many of the signals required for Th17 differentiation and promote the activation of latent transformation growth factor (TGF)-β via αvβ8 integrin [124–126]. Activation of TGF-β is an important requirement for non-pathogenic Th17 differentiation. Regulation of the C-type lectin dectin-1-mediated type I IFN response in human DCs allows TGF-β activation and promotes a nonpathogenic Th17 cellular immune response during fungal infection [127]. In mouse models of lung infection with *Aspergillus fumigatus* or in intestinal infection with *Citrobacter rodentium*, IL-23 that is specifically produced by cDC2s, can help establish an effective Th17 response [32, 115].

CD4^+^ T follicular helper (Tfh) cells support the germinal center response and maintain a prolonged humoral immune response [128–130]. A reduction in pre-Tfh cells was observed in Notch2^−/−^, irf4^−/−^ and other cDC2s-specific deletion mouse models; these mice had impaired humoral immunity and reduced germinal center B cells[131, 132]. Therefore, cDC2s are necessary and sufficient for Tfh cell induction. In addition, DCs enhance Tfh cell differentiation via high expression of CD25 (IL-2 receptor alpha chain) and quenching of T cell-derived IL-2, a potent inhibitor of Tfh differentiation[133]. cDC2s and CD14^+^ macrophages in human tonsils synergistically induce Tfh cell polarization and effector molecule production[134]. Although the absence of DCs results in a slight reduction in Treg numbers, their presence is essential for maintaining the homeostatic proliferation of Tregs [135–137]. The dynamic equilibrium between Tregs and DCs is discussed in the next subsection. Activated CD4^+^ T cells optimize the cellular immune response by enhancing CD8^+^ T cell clonal expansion and differentiation, and DCs may act as a bridge in this process [138–140]. DCs that interact with CD4^+^ T cells can activate antigen-specific CD8^+^ T cells, even in the absence of CD4^+^ T cells after the interaction [141]. In contrast, recognition of cognate antigens on DCs by CD4^+^ T cells induces the production of CCL3/4, which attracts CD8^+^ T cells via CCR5 to recognize antigens on CD4^+^T-DC pairs [142]. The CCR5 ligand also attracts pDCs to the vicinity of CD8^+^T-DC pairs, with subsequent pDC-derived type I IFN production, further promoting the functions of cDC1s [143]. Takagi et al. determined that pDCs inhibit the induction of CD4^+^ T cell responses and are involved in the initiation of CD8^+^ T cell responses under antigenic stimulation and microbial infection [144]. In contrast, antigen-specific CD8^+^ T cell responses were reduced when pDCs were depleted, suggesting an active role for pDCs in cross-initiating CD8^+^ T cells [144]. Indeed, under certain conditions, pDCs can transfer antigens to cDC1s by producing pDC-derived exosomes(pDex), which enable cross-priming of CD8^+^ T cells [145]. Similarly, deficiency in cDC1s results in reduced numbers and function of memory CTL in immunized mice [146].

Altogether, these data suggest that multiple DC subpopulations orchestrate T cell differentiation in vivo and that infection, immunity, and location determine the ultimate polarized fate of T cells. Interestingly, the functional dichotomy between DC1s and DC2s is not invariant and can change in certain inflammatory settings wherein DC2s acquire the ability to activate CD8^+^ T cells. In a recent study, an IFN-I-induced activation state of novel cDC1s was identified, which, unlike the conventional cross-presentation of cDC1s, can acquire and present intact tumor-derived peptide-MHC I complexes, thereby promoting a specific CD8^+^ antitumor response [147].

### **DCs establish central and peripheral tolerance**

Several studies have highlighted that DCs can induce negative selection of thymocytes in vivo, rather than positive selection, to maintain central tolerance [148, 149]. Medullary thymic epithelial cells (mTECs) express many tissue-specific antigens to avoid tissue-specific T-cell production. Thymic DCs can either directly present or cross-present autoantigens that are shed from mTECs and kill T cells if the peptide-MHC complex is strong [150]. Even peripheral DCs can migrate into the thymic medulla to induce self-reactive thymocytes and the formation of naturally occurring Tregs [151]. Circulating DCs can be recruited to the thymic medulla with the assistance of p-selectin, integrin VLA-4 interacting with its ligand VCAM-1, and pertussis toxin-sensitive chemoattractant signaling [149]. Husein et al. also demonstrated that peripheral pDCs can transport antigens to the thymus in the absence of TLR signaling in a CCR9-dependent manner to promote the central negative selection of T cells [152]. However, constitutive deletion of DCs did not affect the normal T cell pool of mice, and central tolerance was not disrupted under steady-state conditions, suggesting that DCs may not be critical for the establishment of central tolerance [135, 153].

Although central tolerance is closely coordinated by multiple mechanisms, it has certain dysregulations. For example, some auto-reactive T cells escape negative selection and some harmless antigens can not be expressed in the thymus [150]. To avoid the resulting immune homeostatic dysfunction, the second layer of peripheral tolerance in the organism is crucial. DCs are similar to sentinels that patrol the body and are widely distributed in peripheral tissues including the skin, kidney, lung, and respiratory mucosa, where they exhibit greater self-tolerance [154]. Relative to normal subsets of DCs, tolerogenic DCs (tolDCs) exhibit lower cross-presentation capacity and lower expression of co-stimulatory molecules [155]. A recent study reported that the Tim-3 bridging protein Bat3 acts as an endogenous regulator of tolDC function[156]. Mechanistically, the lack of Bat3 in DCs leads to enhanced steroidogenesis and suppresses Th1, Th17, and CTL cell responses in a paracrine fashion, thereby diminishing autoimmunity [156]. A tolerogenic subpopulation of DCs (DC-10) that stably express CD163, CD141, CD14, and CD16, produces IL-10 in the absence of IL-12 and enables the induction of T regulatory type 1 (Tr1) cell differentiation, was identified in human peripheral blood and spleen [157].

**Fig. 3:**
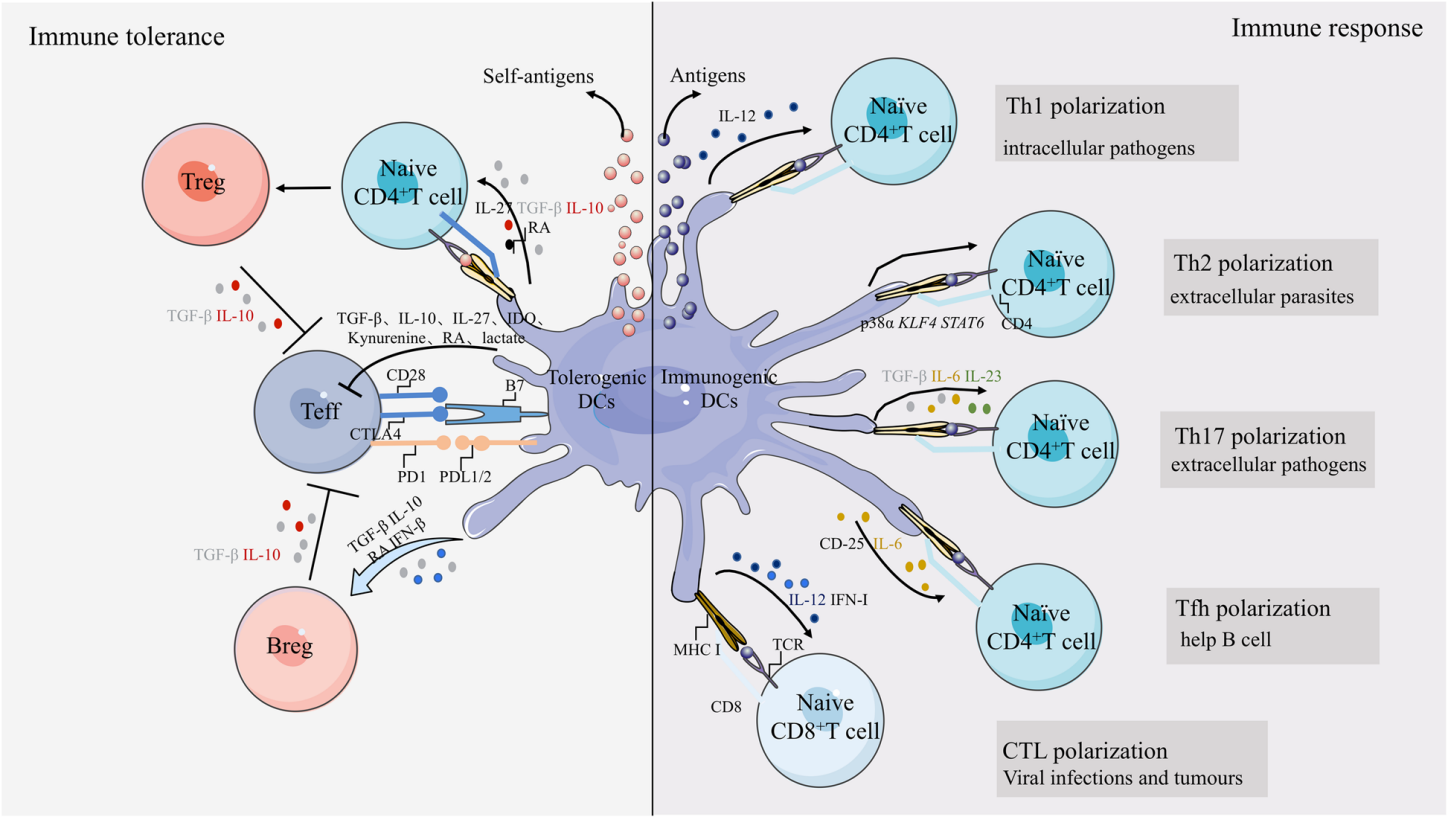
The role of DCs in the induction of immune tolerance and immune response. After activation of DCs by antigens from pathogens or with danger signals, DCs integrate multiple factors to induce T cell polarisation. As an important source of IL-12, DCs promote Th1 cell polarisation. The specific DCs-derived factors that promote Th2 differentiation are unclear, but the transcription factor KLF4 and the STAT6 and p32α signalling pathways play an important role in this pathway. DCs-derived IL-6 is involved in the differentiation of Tfh cells, and in the presence of DCs-derived IL-23 together with TGF-β induces Th17 differentiation [130]. DCs promote the proliferation and function of antigen-specific CTL through antigen cross-presentation, co-stimulatory signalling and derived cytokines such as IFN-I and IL-12 [4]. DCs promote the proliferation and function of antigen-specific CTL through antigen cross-presentation, co-stimulatory signalling and derived cytokines such as IFN-I and IL-12. tolDCs induce immune tolerance in the body by direct or indirect means. PDL1/2 is expressed on most tolDCs, and binding to PD1 on T cells induces T cell apoptosis. In addition, tolDCs secrete a variety of cytokines or metabolites with tolerance activity, such as TGF-β, IL-10, IL-27, Kynurenine, RA and lactate, which tend to promote differentiation of Treg and Breg, creating further suppression of Teff

At steady state, tolDCs have a quiescent or semi-mature nature, in which they trap and process antigens to promote T cell anergy and Treg expansion rather than inducing T cell activation [154]. Several mechanisms underlying the induction of peripheral tolerance by DCs have been identified in mice and humans (Fig. 3). First, cytotoxic T lymphocyte-associated antigen 4 (CTLA4) expressed on T cells competitively binds to B7 molecules on DCs, inducing T-cell anergy [158]. Second, many tolDCs express programmed cell death-ligand 1 (PD-L1 and PD-L2), which induces the resting state of T cells upon binding to programmed cell death protein 1 (PD-1) expressed on T cells[159]. In addition to direct contact with T cells, DCs can induce peripheral tolerance through the secretion of cytokines and metabolites including TGF-β, IL-10, IL-27, indoleamine-2,3-dioxygenase (IDO), kynurenine, and retinoic acid[5]. The roles of the regulatory cytokines IL-10 and TGF-β in Treg proliferation and development have been widely demonstrated [160]. TolDCs express the IDO, rate-limiting enzyme that promotes T-cell apoptosis by blocking the cell cycle of T cells [161]. Notably, IDO is not constitutively expressed in DCs but requires induction by multiple mediators (e.g., TGF-β, endotoxin, TNF, IL-1, and IFN-γ) [55, 161, 162]. Moreover, IL-27 suppresses Th1, Th2, and Th17 cell-mediated immune responses and limit the development of central nervous system (CNS) autoimmunity [163, 164]. In addition, IL-27 regulates CD39 on DCs and Tregs and induces Tr1 cells capable of producing IL-10 [165]. The development of acute graft-versus-host disease is exacerbated by impaired Treg suppression and reduced Tr1 numbers in mice with a deficiency of the p28 subunit of IL-27 in DCs [166]. Furthermore, a clinical trial using human autologous tolDCs for kidney transplantation demonstrated that DCs can shape the T cell response to tolerance by producing high levels of lactate, which is a novel mechanism identified for the suppression of T cell immunity by tolDCs [167].

Similar to Tregs, regulatory B cells (Bregs) are functionally involved in the production of tolerance, highly expression IL-10, TGF-β, and IL-35, and can stimulate T cell anergy as well as regulate invariant natural killer T cell responses [168, 169]. Although Bregs are normally triggered by inflammatory signals, they appear to be induced by DCs during the maintenance of peripheral tolerance [5]. DC-derived IFN-β and CD40 ligands induce the differentiation of mouse spleen B cells into CD19^hi^FcγIIb^hi^ Bregs, which inhibit the CD4^+^ T cell response by secreting IL-10 [170]. In a group of EAE mouse models, antigen-specific vitamin D3 (vitD3) tolerogenic DC cell therapy halted disease progression by inducing multiple tolerance mechanisms, including increased Bregs, reduced natural killer (NK) cells, and immunomodulatory NKT activation [171]. These findings show that not only Tregs but also Bregs, play a role in the maintenance of peripheral tolerance by DCs.

### Promotion of antitumor immunity by DCs

DCs are capable of initiating antigen-specific T cells to generate robust and durable T cell-driven immune responses; therefore, they are known as key regulators of antitumor responses. The tumor microenvironment (TME) can evolve a variety of immunosuppressive mechanisms that impede the effectiveness of DC maturation or DC-induced immune responses, enabling tumors to thrive under unfavorable conditions [172, 173]. Although most tumors contain only a small number of mature DCs, the presence of these DCs is positively associated with improved survival and prognosis in mouse tumor models as well as patients with cancer [94, 174]. The accumulation of DCs in the TME depends on the local production of growth factors and chemokines that promote the recruitment and expansion of DCs [1]. Subsequently, these DCs transport tumor antigens into draining lymph nodes (DLN) in a CCR7-dependent manner; this transport is critical for the initiation of anti-tumor T cells [175–177]. In the absence of CCR7, antitumor T cells in the DLN are no longer efficiently stimulated despite loading of DCs with tumor antigens [176]. NK cells produce XCL1, XCL2, and CCL5 chemokines, which contribute to the recruitment of DCs to the TME; in contrast, FLT3L expressed by NK cells can further promote the differentiation and expansion of DCs [178, 179]. PGE2-mediated tumor immune escape is partly because of the direct inhibition of NK cell production of CCL5 and XCL1 by PGE2, which impairs the localization and accumulation of cDC1s in the TME [178]. Several recent studies have highlighted the potential contribution of microbiota-derived PAMP in counteracting tumor immunity via the activation of DCs [1, 180, 181]. In addition, microbial-derived STING agonists (e.g., c-di-AMP) induce IFN-I production via monocytes in the TME to regulate crosstalk between NK cells and DCs, thereby enhancing the efficacy of immune checkpoint blockade [182]. In turn, the absence of these signals polarizes the differentiation of mononuclear phagocytes to tumor-promoting macrophages [182].

Given that CD8^+^ T cells are often considered the primary effector of antitumor immunity, promoting effective tumor-associated antigen presentation by DCs has been the focus of cancer immunotherapy. The number and stimulatory phenotype of cDC1s in draining lymph nodes has been reported to decrease with tumor progression, and restoration of the cDC1s axis with Flt3L/anti-CD40 treatment resulted in the expansion of tumor-specific CD8^+^ T cells and a reduction in tumor burden [183]. Consistent with this, Batf3^−/−^ or Cd207^cre^Irf8^fl/fl^ mice have reduced numbers of tumor-infiltrating CD8^+^ T cells owing to the lack of cDC1s, thereby promoting the progression of lung adenocarcinoma and fibrosarcoma [21, 184]. The expression of β-linked proteins in cancer cells leads to a reduction in CCL4 concentration in tumors, which prevents CCR5-mediated recruitment of cDC1s and impedes the normal activation of CD8^+^ T cells [185]. Interestingly, selective deletion of MHC II in cDC1s can prevente early activation of CD4^+^ T cells by tumor-derived antigen [186]. This suggests that in the presence of tumor-derived antigens, CD40 signaling in cDC1s is critical not only for CD8^+^ T cell initiation but also for early activation of naïve CD4^+^ T cells.

In contrast to cDC1s, cDC2s present tumor-associated antigens directly to CD4^+^ T cells or transfer them to lymphoid tissue-resident DCs [29, 187]. In oropharyngeal squamous cell carcinoma models, cDC2s are required for Th1 cell polarization and the production of high levels of IL-12 and IL-18 [188]. Tumor infiltration by cDC2s is also positively correlated with infiltration by Tbet^+^ and tumor-specific T cells [188]. In a mouse model of degenerative fibrosarcoma, cDC2s expressing IFN-stimulated genes can acquire and present intact tumor-derived peptide/MHC class I complexes that activate antitumor CD8^+^ T cell responses [147]. Despite the ability of pDCs to produce large amounts of IFN-α/β in the activated state, their specific role in tumors remains controversial [189]. Activation of pDCs in tumor-draining lymph nodes or the presence of TGF-β results in the constitutive expression of the IDO immunosuppressive enzyme, which induces immune tolerance and facilitates tumor progression [190, 191]. moDCs can act as a source of IL-12 to induce a protective Th1 response mice; however, their ability to produce IL-12 is much lower than that of cDC1s [192]. In contrast, moDCs exert immunosuppressive functions in tumors through the production of nitric oxide, a potential T-cell suppressor molecule [193]. The heterogeneity of moDCs may explain their conflicting roles in tumors. Furthermore, different tumor models with different immune backgrounds may contribute to this discrepancy. In the TME, DCs communicate not only with immune cells but also with cancer cells through multiple pathways. For example, tumor-derived VEGF can inhibit FLT3L-mediated activation of NF-κB and negatively affect the production and function of cDCs in vivo [194]. Thus, different subsets of DCs coordinate the body’s antitumor immunity by interacting with multiple cell types (Fig. 3).

## Immunotherapy based on DCs and their signaling pathways

The ability of DCs to induce immune tolerance and adaptive immune responses makes them an optimal candidate for research in cellular immunotherapy. Numerous attempts have been made to exploit the potential of DC-based immunotherapies, from suppressing autoimmune diseases and establishing transplant tolerance to inducing antitumor immunity[195–198].

### The role of DCs in autoimmune diseases and therapeutic perspectives

As previously mentioned, DCs play a double-edged sword role in autoimmune diseases. Prolonged stimulation by autoantigens induces DC activation and maturation, which subsequently initiates self-reactive T and B cells, which disrupts immune tolerance and sustains tissue inflammation [199, 200]. An increase in the maturation phenotype of DCs, such as upregulated expression of co-stimulatory molecules, such as CD86, and increased secretion of pro-inflammatory cytokines, such as IL-12 and IFN-I, have been documented in systemic lupus erythematosus (SLE) patients [201, 202]. Additionally, pDCs isolated from patients with rheumatoid arthritis (RA) secrete higher levels of IFN-α than those from healthy donors [203]. The altered distribution or function of DCs are also part of the pathogenesis of autoimmune diseases [204, 205]. Compared to healthy individuals, patients with autoimmune diseases have a higher distribution of DCs at pathological sites and a lower distribution in the blood [206–208]. Specifically, pDC infiltration that is nearly completely absent in healthy tissue has been observed at significantly higher levels in various pathological tissues, such as the skin of patients with SLE, and muscle tissue of patients with idiopathic inflammatory myopathies [207, 209–211]. This may be attributed to the increased migration ability of DCs in the pathologic state. For example, an increased CCR7 expression in DCs of patients with SLE promotes enhanced migration of CCR7-dependent DCs to the skin [212, 213]. In addition, elevated myeloid growth factors, including IL-3 and GM-CSF, contribute to the inflammatory pathways and pathology of SLE [214, 215]. Activation of the IL-3- or GM-CSF-induced JAK2-STAT5 signaling pathway is necessary to establish mTORC1 activity, which is required for IFN-I production by pDCs at nephrotic sites in patients with SLE [216].

In EAE, DC signals induce pathogenic T cell development following the interaction between prostaglandin D2 (PGD2) and its receptor PTGDR [217]. Mitogen-activated protein kinase p38α regulates the expression of cytokines and co-stimulatory molecules in DCs and promotes pathogenic Th17 differentiation and progression of EAE by interacting with the IL-23 receptor (IL-23R) [218]. The intrinsic IFN-γ-JAK1-STAT1 signaling pathway in DCs promotes the expression of the co-inhibitory molecule PD-L1 and limits T cell proliferation, which is essential for iTreg production and peripheral tolerance during EAE [219]. Furthermore, a recent study showed that optineurin in DCs inhibits the downstream transcription of IL-10 by suppressing JAK2-STAT3 interactions, and inhibition of STAT3 phosphorylation reportedly facilitates the progression of EAE in mice [220]. A mouse model of psoriasiform dermatitis demonstrated that the production of IL-36 by skin cells drives the pathological IL-23/IL-17/IL-22 axis through DCs to promote disease progression, whereas deletion of CD11c^+^ DCs inhibits disease progression [221]. IL-17 A downregulates protein phosphatase 6 (PP6) expression in psoriatic keratinocytes, indirectly promoting TLR7-dependent RNA sensing and IL-6 production by DCs, which subsequently drives the hyperproliferation of keratinocytes [222]. Single-cell analysis of lesional and non-lesional skin from patients with psoriasis by Nakamizo et al. revealed a significant enrichment of CD14^+^ DC3s in damaged skin; these cells are one of the key cell types co-expressing IL-1B and IL-23 A, which are two cytokines critical for psoriasis pathogenesis [223]. In contrast, DCs with immunologic tolerance properties can suppress inflammation by inducing Treg or Breg production and, consequently, inhibit the activation of self-reactive T and B cells [200, 204]. In conclusion, prior data from mouse models and humans suggest that DCs, especially pro-inflammatory DCs, are important in maintaining the activation and polarization of effector T-cells in autoimmune diseases.

**Fig. 4:**
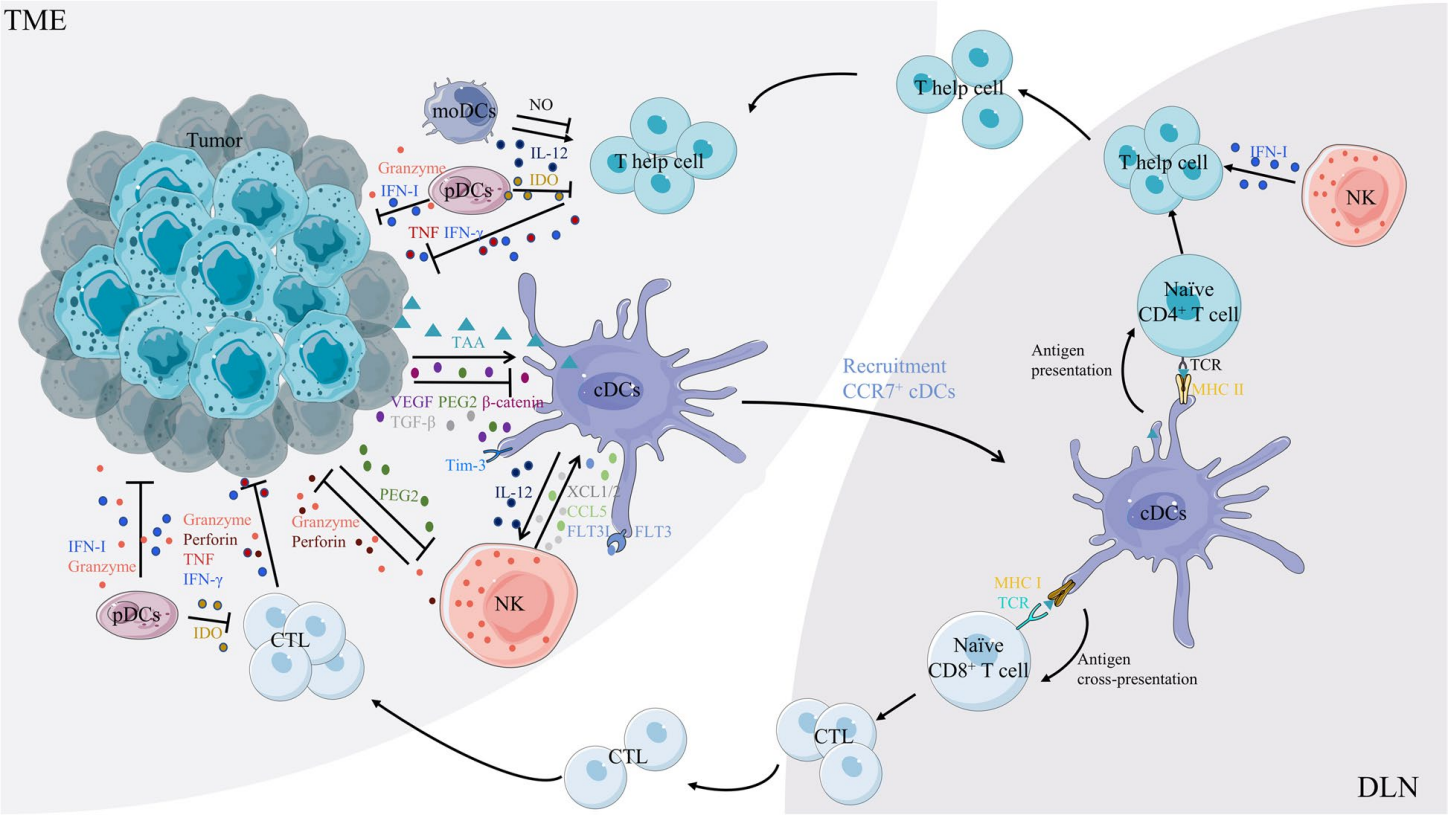
The role of DCs subsets in the tumor microenvironment. The antitumor effects of DCs start with the uptake of TAAs in the tumor microenvironment, followed by the activation and migration of DCs into the DLN. At the same time, tumors interfere with the maturation, recruitment and function of DCs through a series of signals such as secretion of VEGF, PEG2, β-catenin, TGF-β, and upregulation of Tim-3 expression of DCs. DCs migrating into the DLN deliver TAAs to naive T cells via cross-presentation and direct presentation, which promotes differentiation of tumor-specific T helper cells and CTL. There is also an interaction between DCs and NK cells, with IL-12 secreted by DCs promoting the activation and proliferation of NK cells, while NK cells further promote the recruitment and expansion of DCs via XCL1/5, CCL5 and FLT3L. pDCs can produce IFN-I and Granzyme to kill tumor cells, but can also inhibit the killing effect of effector T cells by secreting IDO. pDCs can produce IFN-I and Granzyme to kill tumor cells, but can also inhibit the killing effect of effector T cells by secreting IDO. moDCs exhibit dual effects on CD4^+^ T cells via NO and IL-12. TAAs. tumor- associated antigens; VEGF, vascular endothelial growth factor

Currently, two main types of therapies for autoimmune diseases target DCs (Fig. 4). One approach is systemic immunomodulation by small-molecule drugs or monoclonal antibodies that selectively target a specific function of DCs, including cytokine secretion, antigen processing, antigen presentation, and migratory capacity, or deplete a subpopulation of pro-inflammatory DCs that are critical for the disease pathogenesis [208]. The second approach involves tolDCs with immune tolerance, which are induced in vivo or in vitro. These tolDCs usually exhibit an immature or semi-mature phenotype with low expression of co-stimulatory molecules, reduced secretion of inflammatory cytokines, and increased secretion of anti-inflammatory cytokines [224]. The first drug targeting the co-stimulatory signal between DCs and T cells was the fusion protein CTLA4-Ig, known as abatacept, which has been approved for the treatment of RA with successful results [225, 226]. Several clinical trials targeting pDC for the treatment of SLE have achieved favorable results [227]. VIB7734, a monoclonal antibody targeting the pDC-specific marker immunoglobulin-like transcript 7(ILT7), rapidly and efficiently depletes pDCs in vivo, and the reduction in pDCs in the skin correlates with reduced local IFN-I activity and alleviates clinical disease presentations [228]. B-cell lymphoma 2 (Bcl-2) is critical for pDC survival and IFN-I production in lupus mice or patients with SLE, and the use of the selective Bcl-2 inhibitor venetoclax (ABT-199) significantly reduces auto-reactive B cell and total lymphocyte counts and alleviates disease symptoms in female patients [229, 230]. Inflammatory cytokines secreted by DCs are involved in the hyperactivity of T and B cells as well as tissue damage and are positively associated with disease activity in a range of autoimmune diseases, including type 1 diabetes (T1D), SLE, and RA. Therefore, the inhibition of the action of these cytokines is an important strategy for treating the disease. Tocilizumab blocks the binding of IL-6 to the IL-6 receptor, and its safety and efficacy have been demonstrated in clinical trials of RA, juvenile idiopathic arthritis, and SLE [231, 232]. Etanercept has also been used as an TNF-α blocker in the treatment of many autoimmune diseases, including T1D, psoriasis, and ankylosing spondylitis [233]. In addition, treatment of humanized CCR7 mice with the anti-human CCR7 monoclonal antibody 8H3-16A12 produced favorable prophylactic and therapeutic effects against arthritis [234].

Although immunosuppressive drugs used to treat autoimmune diseases are effective in improving disease outcomes, their use is often accompanied by the risk of chronic infection or cancer development [235]. In this context, tolDCS-based therapy is a promising alternative that specifically abrogates pathological autoimmune or inflammatory responses without compromising protective immune responses against other pathogens and malignancies [236]. Not only DCs themselves, but also various immunomodulatory Dex that are produced by DCs and modified with anti-inflammatory molecules, such as IL-10, IL-4, TGF-β, IDO and CTLA4, have shown promising results for the treatment of immune diseases and chronic inflammation [237–241]. Many small molecules such as dexamethasone, acetylsalicylic acid, minocycline, and vitamin D3, induce tolDC formation [242]. Moreover, dexamethasone and vitamin D3 similarly prevent the maturation of DCs in humans and mice in vitro, leading to a tolerogenic phenotype with downregulation of co-stimulatory molecules and reduced IL-12 production [243, 244]. Rapamycin, IL-10, and GM-CSF have been incorporated into protocols for the in vitro generation of human monocyte-derived tolDCs [5]. Many clinical trials have been performed to assess the feasibility of putative tolDC therapies for the treatment of autoimmune and other inflammatory diseases following the development of these protocols, and to increase understanding of DC-induced self-tolerance (Table 1). In a clinical trial evaluating the safety and tolerability of autologous tolDCs in Crohn’s disease (CD), intraperitoneal administration of tolDCs led to clinical improvement and good treatment tolerability in one-third of the patients [245]. Phase I clinical trials using tolDCs pulsed with proinsulin peptide that were administered intradermally to treat patients with T1D, have also confirmed the antigen-specific immunomodulation of tolDCs as a therapeutic option [246]. In addition, several clinical trials based on tolDCs in multiple sclerosis, RA, and organ transplantation have demonstrated encouraging results [197, 247]. However, significant challenges remain in the large-scale production of protocols for tolDCs with different phenotypes and tolerabilities, as well as in the dose and route of administration for different patients.

**Table 1:** Clinical trials of DCs-based immunotherapy in autoimmune diseases and tumor

Indication	Phase	Status	Treatment	Patient number	NCT Number
**Autoimmune diseases**					
MS	I	Completed	Intradermal or intracolonic administration of tolDC	9	NCT02618902
MS	I	Recruiting	Intracervical lymph node administration of tolDC-VitD3	16	NCT02903537
MS	II	Recruiting	Tol-DC is administered in combination with immunomodulatory drugs	45	NCT04530318
RA	I	Completed	Tol-DCs are administered at the knee joint	15	NCT01352858
RA	I	Completed	Tol-DCs are administered at the knee joint	10	NCT03337165
T1D	II	Unknown	BM-derived DCs are treated with antisense oligonucleotides targeting CD80, CD86, CD40 and then administered intradermally	24	NCT02354911
T1D	I	Recruiting	TolDCs loaded with Proinsulin Peptide (C19-A3) are administered	7	NCT04590872
SLE	II	Active, not recruiting	VIB7734, a drug targeting pDCs, was administered	214	NCT04925934
**Cancer**					
GBM	III	Active, not recruiting	GBM lysate-pulsed autologous DC vaccine was administered intradermally	348	NCT00045968
GBM	I	Completed	GBM lysate-pulsed autologous DC vaccine was administered intradermally	26	NCT01006044
Melanoma	I/II	Completed	Autologous TLR-ligand matured DC vaccine encoding mRNA electroporation of tumor-associated antigens was administered	20	NCT00940004
Melanoma	III	Active, not recruiting	DC vaccines loaded with autologous tumor RNA are administered intravenously	200	NCT01983748
Renal Cell Carcinoma	II	Recruiting	DC vaccines loaded with autologous tumor antigens in combination with immune checkpoint inhibitors are administered	120	NCT04203901
Breast cancer	I/II	Completed	HER2 peptide-pulsed cDC1s vaccines are administered	58	NCT02061332
Ovarian carcinoma	I/II	Not yet recruiting	moDC vaccines loaded with patient-specific peptides or tumor lysates are administered	16	NCT05714306
Prostate Cancer	II	Completed	DC vaccines loaded with tumor antigens in combination with docetaxel are administered	43	NCT01446731
Bladder cancer	I	Recruiting	Anti-CD40 antibody 2141-V11 targeting DCs was administered by intravesical drip	25	NCT05126472

### DCs in cancer therapy

**Fig. 5:**
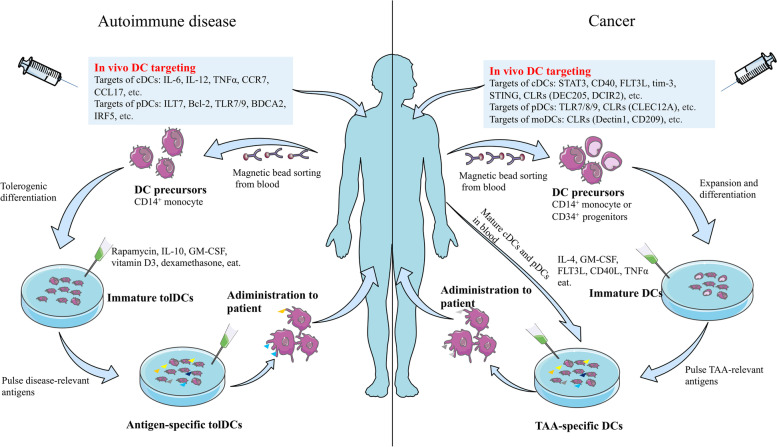
DCs-based immunotherapy. Current strategies for DCs-based treatment of autoimmune diseases and tumors fall into two broad categories. One is the direct targeting of DCs in vivo. In the treatment of autoimmune diseases, the targets of various DCs (e.g., IL-12, Bal-2, ILT7, CCR7) are usually used to induce immune tolerance or suppress their immune response. In the treatment of tumors, DCs are usually targeted by various DCs (e.g., TLRs, CLRs, STING, CD40) to enhance or restore the function of DCs in order to achieve anti-tumor immunity. Another strategy is vaccination of DCs. Precursors of DCs (or mature cDCs and pDCs) are isolated from patient blood, induced to amplify and differentiate with different cocktails, and DCs are pulsed with relevant antigens to give them antigenic specificity. After a final safety and efficacy assessment, they are injected back to the patient

Over the last two decades, clinical trials of DC-related immunotherapy have focused primarily on tumors, rather than autoimmune diseases, and have shown promising results (Table 1). Therapies targeting DCs focus on enhancing or restoring the function of DCs, increasing their number, or inducing de novo antitumor immunity. The therapies can be broadly categorized into DC vaccines and other DC-related trials (Fig. 5). WNT2 secreted by tumor cells or cancer-associated fibroblasts inhibits DC-mediated antitumor T cell responses via the SOCS3/p-JAK2/p-STAT3 signaling cascade, promoting the malignant progression of solid tumors including colorectal cancer, esophageal squamous cell carcinoma, gastric cancer, and breast cancer [248–251]. The use of an anti-WNT2 monoclonal antibody significantly restored antitumor T cell responses in mouse tumors by increasing active DCs [250]. Furthermore, the STAT3 signaling pathway is equally involved in the downstream transcription of IL-6, IL-10, and VEGF, which can impair the differentiation of DCs and inhibit the production of IL-12 in human moDCs [220, 252, 253]. Local injection of a STAT3 antisense oligonucleotide (CpG-STAT3ASO) activates human DCs and promotes CD8 + T-cell recruitment to tumor sites [254]. Many inhibitors of STAT3, including BP1-102, STX-0119, and HJC0123, have been identified clinically and shown to have pro-apoptotic and antitumor activities against STAT3-overactivated cancer cells [255].

As knowledge of the DC signaling pathway accumulates, strategies to directly target DCs in vivo to enhance antitumor immunity are being increasingly developed. Activation of TLR on DCs induces their maturation and promotes the production of pro-inflammatory cytokines and chemokines; therefore, TLR agonists are also widely used to activate DCs [40]. The TLR7/8 ligand imiquimod has been used to treat non-melanoma skin cancers because of its ability to promote CCL2-dependent recruitment of pDCs and induce the secretion of tumor-killing TRAIL and granzyme B from pDCs [256]. Similarly, the TLR3-specific agonist ARNAX effectively initiates DCs to induce antitumor immunity without causing systemic inflammation in mice [257]. Activation of CD40 on DCs upregulates the secretion of co-stimulatory molecules and IL-12 [258]. A newly developed CD40 agonist antibody, 2141-V11b, has been shown to activate long-term systemic antitumor immunity in cDC1s-driven CD8^+^ T cells in orthotopic bladder cancer models and is now in phase I clinical studies [259]. However, the increased potency of Fc-engineered CD40 antibodies in clinical use is accompanied by increased toxicity, such as cytokine release syndrome and macrophage-dependent hepatotoxicity [260–263]. Therefore, Salomon et al. designed bispecific antibodies that preferentially target CD40 on DCs, increasing their specificity and safety while preserving their effective antitumor activity [264]. In addition, VitE can restore the function of DCs in TME by inhibiting intrinsic checkpoint protein tyrosine phosphatase SHP1 and enhancing tumor antigen cross-presentation by DCs and Dex [265]. Eliminating the ability of DCs to induce tolerance in the TME is another strategy for enhancing the antitumor immunity of DCs. In this regard, four IDO inhibitors are in clinical development, and preliminary findings indicate their antitumor activity [266]. Similarly, the anti-TIM-3 antibody upregulates CXCL9 on DCs and promotes cDC1-mediated antitumor immunity, which has been successfully combined with PD-1/PD-L1 blockade to inhibit tumor growth [267, 268].

DC vaccines aim to provide the immune system with in vitro-trained DCs, which are usually isolated from the peripheral blood of patients, properly activated, and loaded with tumor antigens or tumor lysates, to induce a strong antitumor response [196]. Treatment with personalized neoantigen peptide-pulsed autologous DC vaccine (Neo-DCVac) reportedly promotes the secretion of cytokines, such as IL-12, IFN-γ, and TNF-α in patients with metastatic lung cancer, with a disease control rate of 75% [269]. In a phase 2 study, the use of a DC vaccine electroporated with Wilms’ tumor 1 (WT1) mRNA in patients with acute myeloid leukemia was shown to be a safe and feasible regimen in promoting tumor infiltration of multi-epitope WT1-specific CD8^+^ T cells and effectively prevent or delay cancer recurrence after standard chemotherapy [270]. Owing to the rarity of cDCs or pre-DCs in the blood, moDCs are currently used in most clinical studies. The addition of autologous tumor lysate moDC vaccine (DCVax-L) to standard therapy in patients with glioblastoma (GBM) prolongs patient survival and has a favorable safety profile, with only 2.1% of patients experiencing treatment-related grade 3 or 4 adverse events [271]. However, endogenous DCs are required for the initiation of tumor-specific T cells after moDC vaccination [15]. Owing to their excellent antigen-presenting ability and high specificity, cDCs are considered promising antitumor vaccine candidates [272, 273]. HER2 peptide-pulsed cDC1 vaccines induce specific anti-HER2 CD4^+^ and CD8^+^ immune responses in the treatment of patients with HER2^pos^ tumors, independent of the vaccination route [274]. It is important to note that cDC1s vaccines can directly engage in antigen presentation to drive tumor-specific CD8^+^ T cell responses in mice independent of host cDC1s [275].

## Conclusion and outlook

Undoubtedly, the advances made in DCs research in recent years have led to increased knowledge of many key transcription factors and cytokines that are involved in the regulation of DCs differentiation and function. To capitalize on these discoveries, a growing number of clinical trials based on DCs therapies are being initiated. Some trials have shown promising safety and efficacy in the treatment of autoimmune diseases and cancers. However, considerable challenges remain in establishing effective and applicable DC vaccines. One challenge is ensuring that DC vaccines avoid the effects of immunosuppressive factors in TME, including IDO, IL-10, TGF-β, and PEG2. Moreover, most DC vaccines do not effectively migrate to the lymph nodes to mobilize the T cell immune response. Moreover, DC-based immunotherapies are often expensive and require complex manufacturing processes, which limit access to a wide range of patients.

The primary focus of research on DCs is to identify the precise conditions which determine whether DCs exert their immunosuppressive or immune-activating functions. The diversity of different DCs subpopulations in terms of origin, location, phenotype, and function makes this challenging. Future research should explore the biological properties of DCs in controlling T cell immunity to maximize their therapeutic potential. In addition, continuous advancement in technology has the potential to revolutionize the field of DC-based immunotherapy. The use of nanotechnology may enable the more effective delivery of DC-targeted drugs or vaccines. Advancements in gene editing technologies like CRISPR/CAS9 may enable the development of more effective and specific DC-based therapies. Overall, continued research in the field of DC biology and immunotherapy has the potential to revolutionize the treatment of a wide range of diseases and offers hope to patients with a wide range of disorders.

## Data Availability

Not applicable.

## Declarations

**of interest**.
